# Elucidating the evolutionary history and expression patterns of nucleoside phosphorylase paralogs (vegetative storage proteins) in *Populus* and the plant kingdom

**DOI:** 10.1186/1471-2229-13-118

**Published:** 2013-08-19

**Authors:** Emily A Pettengill, James B Pettengill, Gary D Coleman

**Affiliations:** 1 Department of Plant Science and Landscape Architecture, University of Maryland, Plant Science Building, College Park, Maryland, 20742, USA; 2 Department of Plant Science and Landscape Architecture, University of Maryland, Takoma Park, Maryland, USA

**Keywords:** Nucleoside phosphorylases, Vegetative storage proteins, Bark storage proteins, Nitrogen cycling, *Populus trichocarpa*

## Abstract

**Background:**

Nucleoside phosphorylases (NPs) have been extensively investigated in human and bacterial systems for their role in metabolic nucleotide salvaging and links to oncogenesis. In plants, NP-like proteins have not been comprehensively studied, likely because there is no evidence of a metabolic function in nucleoside salvage. However, in the forest trees genus *Populus* a family of NP-like proteins function as an important ecophysiological adaptation for inter- and intra-seasonal nitrogen storage and cycling.

**Results:**

We conducted phylogenetic analyses to determine the distribution and evolution of NP-like proteins in plants. These analyses revealed two major clusters of NP-like proteins in plants. Group I proteins were encoded by genes across a wide range of plant taxa while proteins encoded by Group II genes were dominated by species belonging to the order *Malpighiales* and included the *Populus* Bark Storage Protein (BSP) and WIN4-like proteins. Additionally, we evaluated the *NP-like* genes in *Populus* by examining the transcript abundance of the 13 *NP-like* genes found in the *Populus* genome in various tissues of plants exposed to long-day (LD) and short-day (SD) photoperiods. We found that all 13 of the *Populus NP-like* genes belonging to either Group I or II are expressed in various tissues in both LD and SD conditions. Tests of natural selection and expression evolution analysis of the *Populus* genes suggests that divergence in gene expression may have occurred recently during the evolution of *Populus*, which supports the adaptive maintenance models. Lastly, *in silico* analysis of *cis*-regulatory elements in the promoters of the 13 *NP-like* genes in *Populus* revealed common regulatory elements known to be involved in light regulation, stress/pathogenesis and phytohormone responses.

**Conclusion:**

In *Populus*, the evolution of the NP-like protein and gene family has been shaped by duplication events and natural selection. Expression data suggest that previously uncharacterized NP-like proteins may function in nutrient sensing and/or signaling. These proteins are members of Group I NP-like proteins, which are widely distributed in many plant taxa. We conclude that NP-like proteins may function in plants, although this function is undefined.

## Background

Nucleotides, nucleotide precursors and derivatives are essential components for life. They compose nucleic acids, act as signaling molecules, intercellular energy transporters, and can be converted to essential enzymatic cofactors. Nucleotide metabolism is therefore a necessary cellular function [1,2]. In mammalian and bacterial systems, nucleoside phosphorylases (NPs) salvage nucleosides by cleaving the glycosidic bond of (deoxy-) ribonucleosides in the presence of inorganic phosphate (Pi) to yield (deoxy-) ribose-1-phosphate and a nucleobase [1,3]. The free nucleobase can be synthesized into organic molecules or degraded, and the (deoxy-) ribose-1-phosphate can be utilized by the Pentose Phosphate Pathway and glycolysis [3]. The most widely studied NPs are purine nucleoside phosphorylases (PNPs), which are a focus in clinical and cancer research for their role in mutation-related immunodeficiency diseases, prostate cancer, leukemia and periodontal disease [4-6].

The ability to salvage purines is particularly important for N-limited plants in the context of retaining and remobilizing nitrogen (N) [7,8]. The proposed physiological role of purine degradation in plants is that it promotes nitrogen-use efficiency (NUE) through mobilization from source to sink [8]. However, there is no evidence that NPs are involved in purine nucleoside salvage in plants; instead hydrolysis of nucleosides occurs by nucleosidases (EC 3.2.2x) [9-11]. Notably, genes in purine salvage and degradation pathways are induced by wounding, drought, abscissic acid (ABA), dark conditions and dark-induced senescence consistent with a role in NUE [12-14]. Although purine salvage appears to be a component of NUE, the role of NP-like proteins in plants in relation to nucleoside salvaging and NUE is not known.

There is a large body of research on a family of proteins that share sequence similarity with PNPs in *Populus* (reviewed by [15]). In this genus, these proteins have an eco-physiological role in seasonal and short-term nitrogen storage. One subfamily of these proteins are termed Bark Storage Proteins (BSPs) since they accumulate in bark parenchyma and xylem rays in autumn and decline in abundance when growth resumes in spring [16-18]. The autumn accumulation of BSP and associated gene expression is a phytochrome mediated photoperiod response [19,20]. In addition, BSPs and related Vegetative Storage Proteins (VSPs) genes are also expressed following wounding, high nitrogen and drought stress suggesting a role in short-term storage [21-23]. Seasonal storage facilitated by BSPs is likely an important evolutionary adaptation that facilitates perennial growth in low nutrient forest systems while short-term storage helps conserve nutrients in response to stresses. This seasonal adaptation uncouples N demands for growth from uptake and assimilation and provides a competitive advantage under conditions of low N supply [24,25]. *Populus* provides a model system to study NP-like proteins and genes not only because of their importance in seasonal and short-term storage but also because *Populus* possesses more NP-like proteins than any other known plant genera.

The involvement of NP-like proteins in seasonal N cycling could result from the expansion and functional evolution of protein and gene families which provides innovation for adaptation and speciation [26,27]. Gene duplication is a mechanism for such innovation and occurs through whole genome duplications (WGD) or small-scale genome duplications such as tandem duplications (TD) [28]. Following duplication, genes can have many fates: duplicates may amplify or buffer original function [29-31], gain a novel function (neofunctionalization) [32,33], accumulate mutations that subdivide the original function (subfunctionalization) [34,35] or become non-functional (pseudogenization) [32]. Because seasonal N cycling is likely an adaptive trait, we considered the main models of adaptive maintenance and gene expansion which are positive dosage, neofunctionalization, subfunctionalization, diversification of multifunctional genes and the dosage balance model (reviewed by [36]). Positive dosage describes the retention of duplicate genes that increase fitness by buffering or functional redundancy [29,30,37]. Neofunctionalization is the gain of new function in the duplicate genes through neutral mutations followed by positive selection while preserving the parent copy [32,33]. The subfunctionalization model suggests that neutral mutations in duplicate genes weaken or alter the original function so that both copies are maintained to perform the original function [35,36]. Under the diversification of multifunctional genes model, a multifunctional parent gene is uncoupled among gene duplicates [38]. The dosage balance hypothesis is useful for explaining the retention of genes following the type of duplication event: the duplicate maintaining the stoichiometric balance of protein complexes, favoring high retention rates for genes and proteins with many interactions [39,40].

The aims of this research were to investigate the phylogenetic and evolutionary relationships of NP-like proteins in *Populus* and the plant kingdom to further our understanding of their functional evolution and the extent to which they may be involved in nutrient salvaging and in particular N cycling and NUE. We first constructed the evolutionary relationships among 13 *NP-like* genes in *Populus*, examined transcript abundance in four tissue-types under long day (LD) and short day (SD) conditions and compared the structure of promoter regions of these genes to gain insight into gene expression and regulation. We further examined the functional divergence of the genes by testing whether any of the genes are under divergent natural selection and when gene expression diverged. Finally, we conducted phylogenetic analyses of NP-like proteins across the plant kingdom. Our analyses of natural selection and the evolution of gene expression patterns were then considered in light of this broader phylogenetic context to draw conclusions about the model of evolution and possible gene fates of *NP-like* genes and proteins within *Populus*.

## Results

### Protein phylogeny and chromosome location of *NP-like* gene family in *Populus*

**Figure 1:**
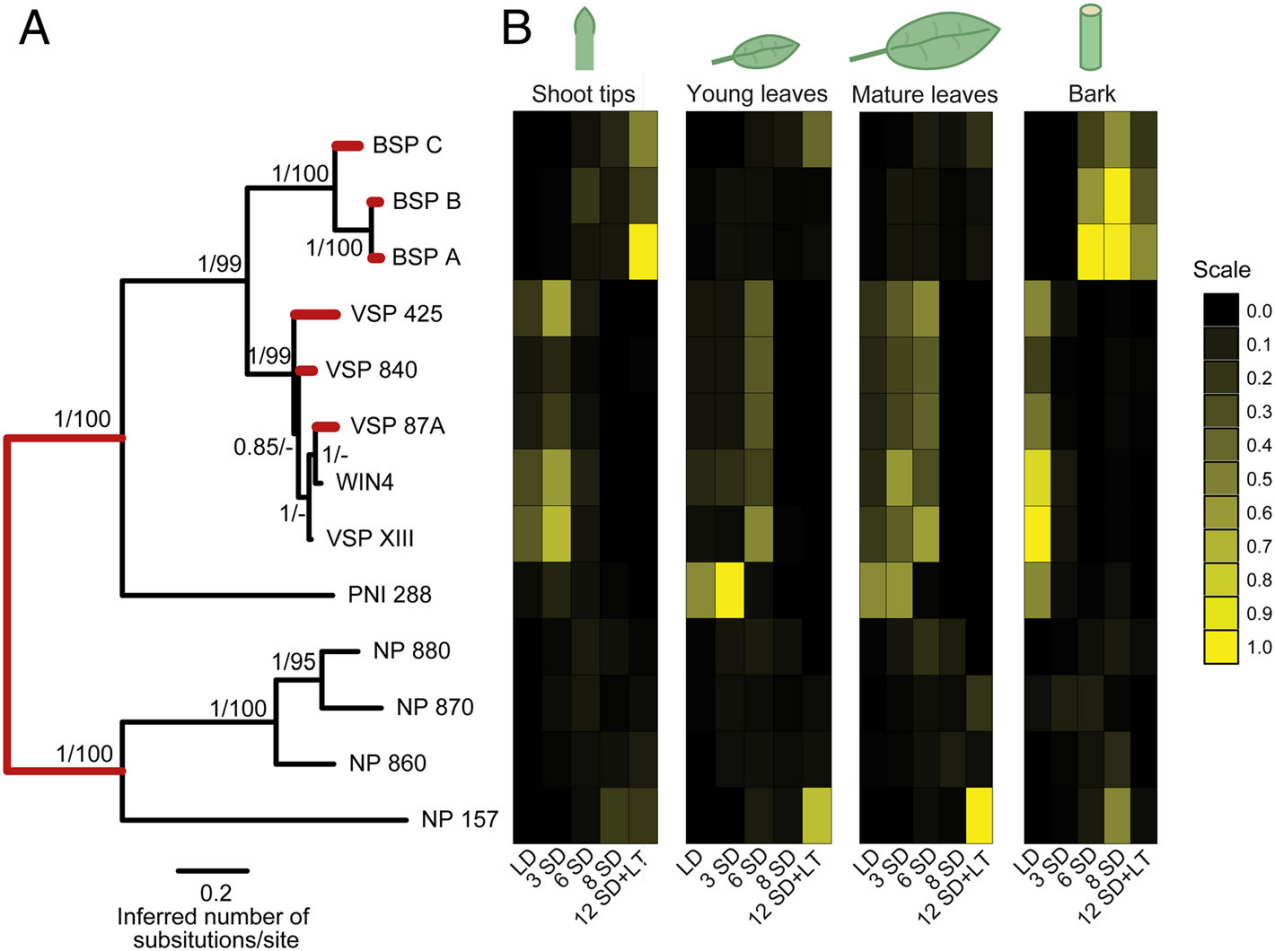
**NP-like family in *Populus trichocarpa. *(A)** Phylogenetic relationship of 13 NP-like proteins in *Populus*. Numbers at branches indicate posterior probabilities and bootstrap percentages based on 1000 replicates, respectively. Branches in red indicate significant evidence for experiencing episodic diversifying selection based on the branch-site REL test implemented in HyPhy. **(B)** Heat map representing the relative transcript expression of *NP-like* genes in shoot tips, young leaves, mature leaves and bark after 8 weeks long-day (LD) conditions and after 3, 6, 8 and 12 weeks short-day (SD) conditions. The 12 week short-day treatment was combined with low-temperature for the final 4 weeks of SD (i.e. after 8 weeks SD). Values were rescaled within each gene between 0 and 1 with 1 indicating highest relative expression levels.

**Figure 2:**
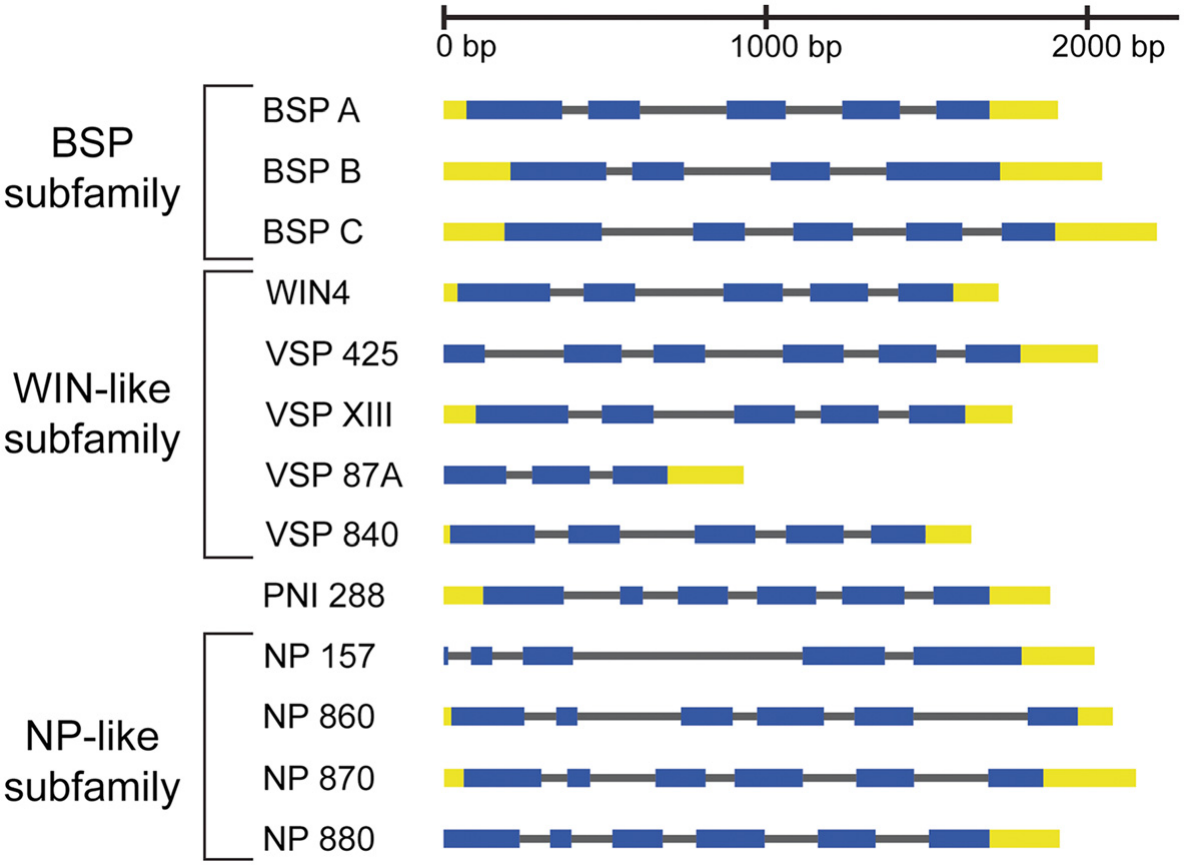
**Intron-exon structure for *NP-like *genes in *Populus trichocarpa*.** A comparison of gene structure was constructed using the primary *NP-like* transcripts from Phytozome (http://www.phytozome.net) and the gene structure draw server found at the PIECE database (http://wheat.pw.usda.gov/piece/index.php). *NP-like* gene subfamilies are indicated by brackets.

To better understand the relationship between the NP-like proteins within *Populus* we constructed an evolutionary tree based on full-length protein sequences retrieved from the *Populus trichocarpa* genome through Phytozome (Table 1). This tree indicates three subfamilies of clustered proteins with strong support, posterior probabilities of 1.0 and bootstrap support over 99% (Figure 1A). The first subfamily is comprised of BSP A, BSP B and BSP C and is designated as the BSP subfamily (Figure 2). The second cluster of proteins includes WIN4 and WIN4-like proteins and is designated as the WIN4-like subfamily. A third subfamily includes four uncharacterized NP-like proteins that we designated as the NP-like subfamily. PNI 288 clustered within the cluster composed of WIN4 and BSP subfamilies, but based on this analysis the protein is distinct from the WIN4 and BSP subfamilies (Figure 1A). Gene structure of the primary *NP-like* transcripts reveals common intron-exon boundaries found within each gene subfamily (Figure 2).

**Table 1:** **List and description of *NP-like *genes within *Populus trichocarpa *used for quantitative gene expression analyses and qPCR primer information**

**Gene symbol**	**Gene name**	**Locus name Phytozome v2.2 (POPTR) and v3.0 (Potri)**	**Primers (5′-3′)**	**Product size (bp)**	**Annealing temp (°C)**	**PCR efficiency**
*BSP A*	Bark Storage Protein A	POPTR_0013s10380	F: TGGAGAGAACTTGTTGGGGAC	81	55	1.981
		Potri.013G100700	R: CAGAAAACTTCCTTGGGCG			
*BSP B*	Bark Storage Protein B	POPTR_0013s10370	F: ATGTTCTCTCCAAGTGAAGCAC	130	57.6	1.981
		Potri.013G082800	R: CGGGCAGGCATTTATCTG			
*BSP C*	Bark Storage Protein C	POPTR_0013s10350	F: TTCGTGGTGTTCCAAGGTG	85	54	1.936
		Potri.013G101000	R: AGGCGTTGTAGGAGGCTAAG			
*WIN4*	Wound Induced 4	POPTR_0423s00200	F: AGGATTTTCGCCTGCTGG	64	62.6	2.032
		Potri.013G080500 and/or Potri.013G082800	R: AATGAACTTGGCTGCGGC			
*PNI 288*	Poplar nitrogen-regulated cDNA 288	POPTR_0019s07690	F: TGCCAATAGATTCAATGCCAC	60	55	1.976
		Potri.019G050200	R: GAAGCCAAAGCAACAGCAG			
*VSP 87A*	WIN-like VSP 87A	POPTR_0013s07850	F: TGAACGGAGAGAACTTGTTGGC R: AGGATGTGGTGCTGGGAAGC	105	54	1.912
		Potri.013G082600				
*VSP 425*	WIN-like VSP 425	POPTR_0013s07810	F: CAAATGTAGCAGGTGAAGCAAG	154	54	1.983
		Potri.013G080400	R: TCAAACGACTCAGAAGCAGATAC			
*VSP XIII*	WIN-like VSP XIII	POPTR_0013s07800	F: TCCAGGATTATCGCCTGCTA	110	55	2.040
		Potri.013G080300	R: AATCCCATCACTCACAAGCC			
*VSP 840*	WIN-like VSP 840	POPTR_0013s07840	F: CCTCCTACAATGCTTTCCTTGCTG	91	55	1.96
		Potri.013G082700	R: GCAGATACAAAATCCCATCACTCAC			
*NP 157*	NP-like 157	POPTR_0006s16610	F: GCTGTAGATGCTTCACTTAGGTTC	65	54.4	1.986
		Potri.T096300	R: GCCTTATTCGGTAGTTCCAAC			
*NP 860*	NP-like 860	POPTR_0008s02860	F: TCAAACGGGTATCCTGTGATTGTC	96	61	2.07
		Potri.008G028500	R: TGCTAAGGGTCCAAATGTCTGG			
*NP 870*	NP-like 870	POPTR_0008s02870	F: AGGGGATGGAACTGGAGAAGTG	102	60	2.01
		Potri.008G028600	R: CCACGAAAATGTCTGCGGTTG			
*NP 880*	NP-like 880	POPTR_0008s02880	F: GCTACCAGGATACAACTCTCCATTG	127	55	1.99
		Potri.008G028700	R: GCTGAAGAACCCCTAAAGATGTCTC			

**Figure 3:**
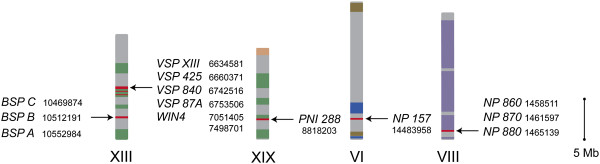
**Chromosome location of *NP-like *genes in *Populus trichocarpa***. Arrows indicate approximate location of genes. Roman numerals indicate chromosome designation and numbers reflect start site location according to Phytozome (http://www.phytozome.net). Colored blocks indicate known syntenic regions within the *Populus trichocarpa* genome v2.0 and the link for these coordinates can be found in the Methods.

The chromosome locations of the genes revealed that the *Populus NP-like* genes comprising the three subfamilies reside on four chromosomes (Figure 3). There are two clusters of *NP-like* genes on chromosome XIII that includes one cluster of all the *BSP* subfamily members and a second cluster that includes the *WIN4-like* subfamily gene members. The *NP-like* subfamily genes are clustered together on chromosome VIII. Only *PNI 288* and *NP 157* were found to not be clustered with other members of the *NP-like* genes with *PNI 288* located on chromosome XIX while *NP 157* is on chromosome VI.

### Expression patterns within the *Populus NP-like* gene family

Gene expression analysis of the *NP-like* gene family in *P. trichocarpa* was assessed in four tissue types (shoot tips, young leaves, mature leaves and bark) of plants treated with LD, SD and SD combined with low-temperatures using qPCR (Table 1 and Figure 1B). Overall, expression of genes within each subfamily was associated with a particular type of tissue and environmental treatment. Expression of all three *BSP*s increased during SD treatment in bark and after SD combined with LT in shoot tips (Figure 1B). Although expression of all three *BSPs* was associated with SD, some differences in expression were observed, notably the induction of *BSP C* in young and mature leaves after 12 weeks SD when the last 4 weeks of SD were combined with low-temperatures. The expression of *BSP A* and *BSP B* were very similar and these two genes are more closely related to each other while *BSP C* is more distant.

In contrast to the *BSP* subfamily where expression was mostly associated with SD and perennial tissues (shoot tips and bark), expression of the *WIN4-like* subfamily members was greater only in bark of LD treated plants and during the early stages of SD photoperiod (3 and 6 weeks) treatment for shoot tips and young and mature leaves. For all the *WIN-4-like* genes, continued exposure to SD resulted in a decline in the steady state abundance of mRNA. This decline in expression of *WIN4-like* genes in plants treated with SD was most dramatic in bark. These patterns of gene expression show that expression of *BSP* subfamily members are closely associated with SD in perennial tissues such as shoot tips and bark while members of the *WIN4-*subfamily are associated with LD in these same perennial tissues. Furthermore, compared to the *BSP* subfamily the *WIN4-*subfamily members are also expressed to a greater extent in both young and mature leaves. *PNI 288* expression was similar to members of the *WIN4-like* subfamily and was detected in all tissues with expression declining during SD treatment.

Except for *NP 157*, expression of the *NP-like* genes (*NP 880*, *NP 870*, *NP 860*) was observed to occur at lower levels in shoot-tips, young leaves, matures leaves and bark when compared to members of the *BSP* and *WIN4-like* subfamilies. Expression of the *NP-like* subfamily genes also tended to be associated with SD conditions in all tissues. *NP 157* was expressed to a greater level than other members of the *NP-like* subfamily and expression was associated with SD in all tissues. The greatest levels of expression for *NP 157* were observed in both young and mature leaves after 12 weeks of SD with the last 4 weeks SD combined with low temperature.

**Figure 4:**
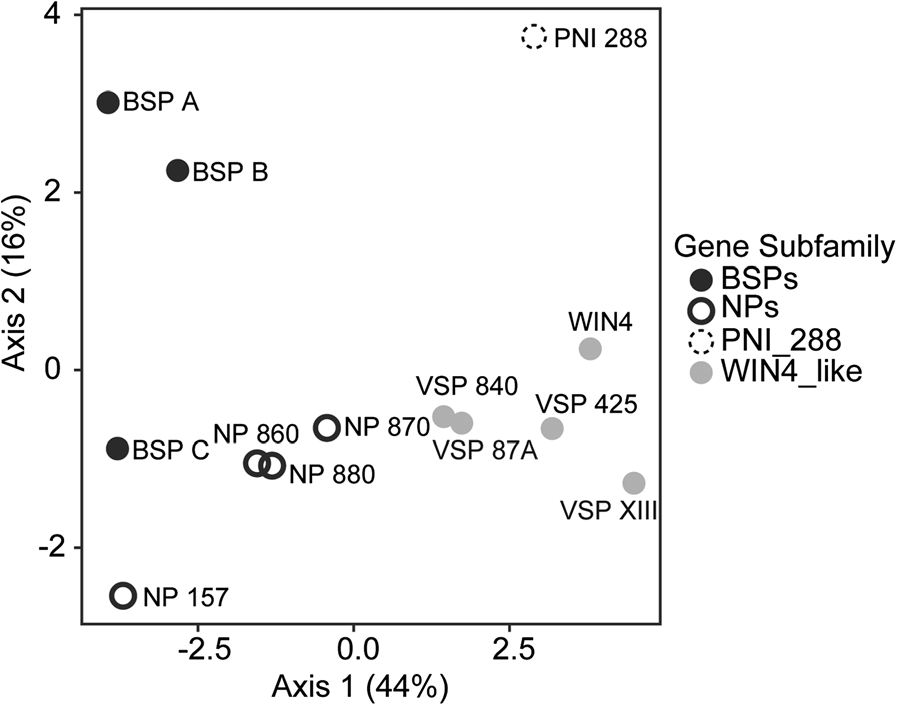
**Principal component analysis (PCA) of *NP-like *gene expression in *Populus trichocarpa*.** PCA illustrating the relationships of *NP-like* genes in *Populus trichocarpa* based upon the expression data presented in Figure 1B*.* The percentage of variation explained by each axis is given in parentheses next along each axis.

In summary, members of each of the three *NP-like* subfamilies (*BSP*, *WIN4-like* and *NP-like*) were observed to have similar expression patterns that were associated with each subfamily. Members of the *BSP* subfamily are expressed in SD and tend to be associated with perennial tissues. Members of the *WIN4-like* subfamily are found in both perennial and deciduous tissues but are repressed by SD. The *NP-like* subfamily is found in both perennial and deciduous tissues but only one member, *NP 157*, appears to show a consistent SD response among the various tissues. Combined with the phylogenetic analysis our analysis of gene expression demonstrates a correlation between phylogenetically defined *NP-like* subfamilies and gene expression. This relationship is also supported by PCA based on the expression data (Figure 4), which shows that members of the same gene subfamily are closer to one another than to members of the other gene subfamilies. Within this analysis, PCA Axis 1 and Axis 2 explained 44% and 16% of the variance, respectively. To determine the number of non-trivial components produced through the PCA, we compared our observed eigenvalues to the distribution created by conducting the analyses on 10000 randomized matrices of the observed dataset (e.g., *λ*_*k*_). Based on this approach, we found that only the first axis had an eigenvalue significantly greater (*P* < 0.05) than that expected by random.

### Natural selection and continuous character evolution

Based on the Branch-site REL method, we found statistically significant evidence for episodic diversifying selection within *VSP 425*, *VSP 840*, *VSP 87A*, *BSP A*, *BSP B* and *BSP C* (Holm-Bonferroni corrected *p*-value < 0.05) (Figure 1A and Table 2). We also found evidence for diversifying selection at the base of the gene tree corresponding to the initial gene duplication that gave rise to phylogenetic clusters referred to as Group I and Group II NP-like proteins (Figure 1A and Table 2).

**Table 2:** Results of tests for a significant phylogenetic signal of the expression data as measured by Pagel's lambda transformation

**Condition**	**λ**_**Bark**_	**λ**_**Shoot tips**_	**λ**_**Mature leaves**_	**λ**_**Young leaves**_
LD	0.658	0.414	0.972**	0.974**
SD 3	0.000	0.502	0.845*	0.986***
SD 6	0.901	0.000	0.792*	0.815*
SD 8	1.000*	1.000*	1.000*	0.000
SD 12	0.936*	0.667	1.000*	1.000*

A value of 0 represents no signal with a value of 1 signifying complete phylogenetic patterning. *P-*values denoted as *P*-value < 0.05, ** *P*-value < 0.01 and *** *P*-value < 0.001.

Tests for whether there is a phylogenetic signal, λ, to gene expression patterns were significantly different than the alternative of no signal (i.e., Brownian motion model of character evolution) (Table 3). Additionally, our tests of evolutionary differences between the NP-like proteins within *Populus* illustrated that expression divergence was concentrated late in the evolutionary process. Specifically, δ was greater than 1 which is indicative of evolutionary changes occurring recently and that a model raising all branches to the power δ was a better fit than a Brownian motion model (Table 3).

**Table 3:** **Results of the tests for episodic diversifying selection among the 13 *NP-like *genes assayed within *Populus trichocarpa***

**Branch**	$\bar{\omega }$	**ω**^**-**^	**Pr[ω = ω**^**-**^**]**	**ω**^**N**^	**Pr[ω = ω**^**N**^**]**	**ω**^**+**^	**Pr[ω = ω**^**+**^**]**	**LRT**	***p*-value**	**Corrected *p*-value**
VSP425	0.911	1.0	0.888	1.0	0.009	10000.0	0.102	120.182	0.0	0.0
VSP840	1.078	0.607	0.968	1.0	0.004	72.780	0.028	12.596	0.0	0.004
VSP87A	10.0	0.0	0.748	0.0	0.071	10000.0	0.180	8.721	0.002	0.033
Node3	1.855	0.0	0.653	0.0	0.052	21.384	0.296	8.193	0.002	0.042
BSPA	0.394	0.279	0.996	0.281	0.0	10000.0	0.004	8.079	0.002	0.043
BSPC	0.899	0.0	0.673	0.0	0.236	12.791	0.091	8.013	0.002	0.042
Node2	0.324	0.202	0.934	1.0	0.002	24.413	0.064	5.244	0.011	0.187
BSPB	0.595	0.059	0.959	0.197	0.001	21.161	0.040	3.666	0.028	0.444
NP880	0.155	0.094	0.959	0.841	0.0	593.764	0.041	2.912	0.044	0.660
NP157	0.545	0.0	0.499	0.857	0.0	2.940	0.501	0.754	0.193	1.0
NP860	0.174	0.108	0.855	0.730	0.120	684.793	0.026	0.587	0.222	1.0
Node12	0.446	0.302	0.881	0.650	0.0	3.380	0.119	0.277	0.299	1.0
Node9	10.0	0.0	0.240	0.0	0.111	10000.0	0.649	0.245	0.310	1.0
Node14	0.375	0.150	0.832	0.993	0.0	2.068	0.168	0.186	0.333	1.0
Node5	0.163	0.0	0.404	0.321	0.554	3333.11z	0.042	0.172	0.339	1.0
PNI288	0.540	0.0	0.428	0.999	0.0	1.192	0.572	0.108	0.371	1.0
VSPXIII	0.973	0.986	0.0	0.984	0.0	1.040	1.0	0.001	0.486	1.0
NP870	0.301	0.165	0.867	1.0	0.060	3.224	0.073	-0.380	0.500	1.0
Node7	0.0	0.0	0.885	0.996	0.0	2.106	0.115	-0.001	0.500	1.0
Node1	10.0	0.093	0.971	0.894	0.028	8.630	0.001	-0.001	0.500	1.0
Node21	10.0	0.095	0.009	0.897	0.010	8.819	0.980	-0.001	0.500	1.0
Node17	10.0	0.054	0.370	0.492	0.232	0.293	0.398	0.0	1.0	1.0
WIN4	0.545	0.714	0.0	0.722	1.0	1.578	0.0	0.0	1.0	1.0

Branch = The name of the branch (see tree plot on the main analysis page for the location of automatically named internal branches).

$\bar{\omega }$ = The ω ratio inferred under the MG94xREV model that permits lineage-to-lineage but no site- to-site ω variation.

ω^-^ = The maximum likelihood estimate (MLE) of the first rate class with ω ≤ 1.

Pr[ω = ω^-^] = The MLE of the proportion of sites evolving at ω-.

ω^N^ = The MLE of the second rate class with ω ≤ 1.

Pr[ω = ω^N^] = The MLE of the proportion of sites evolving at ωN.

ω^+^ = The MLE of the rate class with unconstrained ω.

Pr[ω = ω+] = The MLE of the proportion of sites evolving at ω + .

LRT = Likelihood ratio test statistic for ω + = 1 (null) versus ω + unrestricted (alternative).

*p*-value = The uncorrected *p*-value for the LRT test.

Corrected *p*-value = The *p*-value corrected for multiple testing using the Holm-Bonferroni method.

### *In silico* promoter sequence analyses of the *NP-li*ke gene family in *Populus*

**Figure 5:**
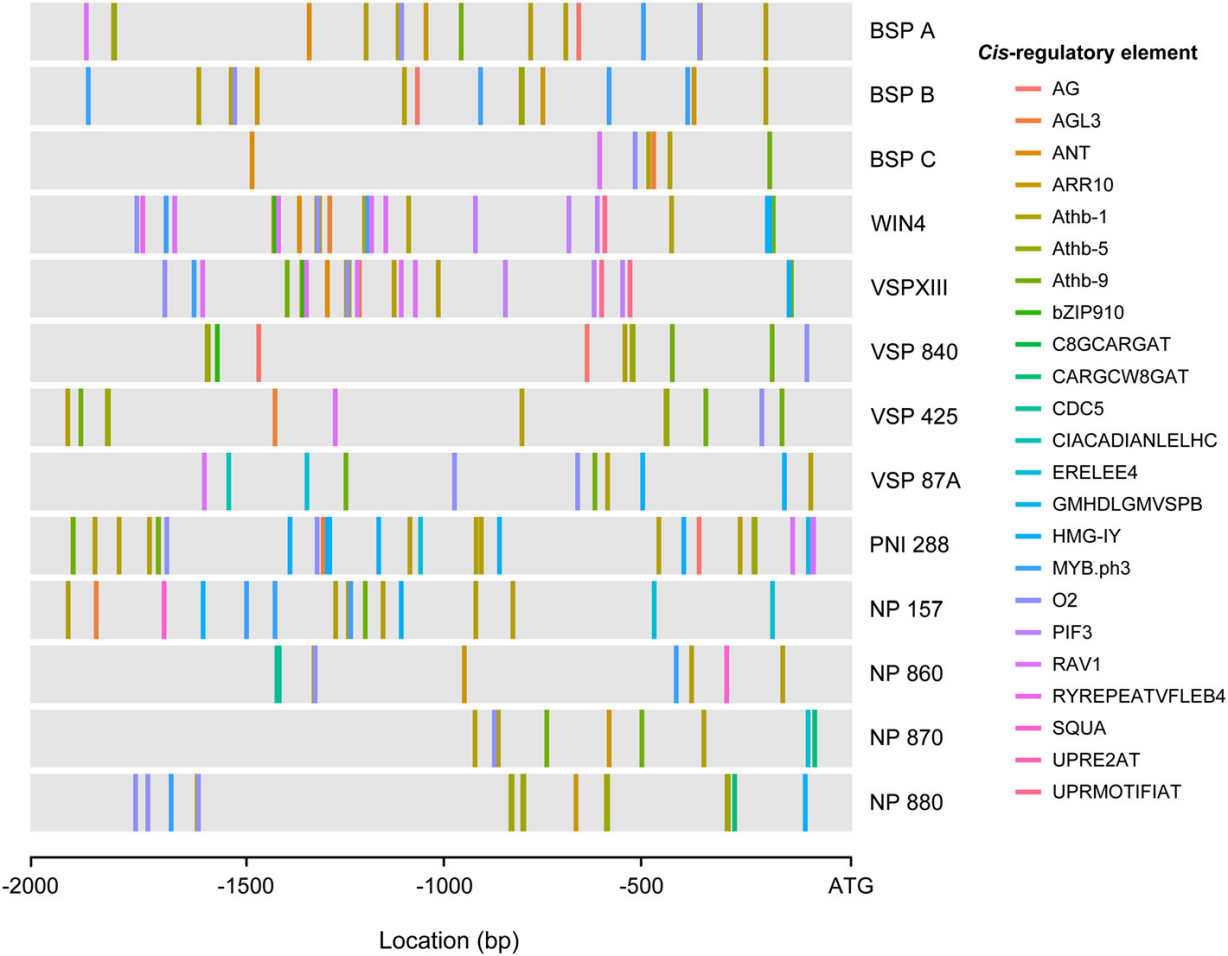
***Cis*-regulatory element distribution in the promoter regions of *NP-like *genes in *Populus trichocarpa*.** CREs identified by two methods of motif prediction are represented as colored bars and correspond to the CRE name.

We identified overrepresented motifs present in the 2 kb regions upstream of *Populus NP-like* genes by performing *in silico* analyses using two prediction programs, MEME and MotifClick [41,42]. We identified motifs that correspond to known *cis*-regulatory elements (CREs) from the transcription factor (TF) database consolidator program PlantPAN [43]. Of the 200 motifs identified by MEME and MotifClick, 23 motifs correspond to known CREs (Figure 5, Table 4, Additional file 1: Table S6). These regions have been shown to bind TFs, which possibly regulate transcription in a range of tissues and in response to phytohormones, stress and light (Table 4). CREs were found in the *Populus NP-like* gene promoters that may contribute to tissue-specific expression (AG, AGL3, Athb-1, -5, -9, CDC5, MYB.ph3, O2 and SQUA), phytohormone responses (ARR10, ERELEE4, RAV1 and RYREPEATVFLEB4), stress responses (RAV1, UPRE2AT and UPRMOTIFIAT) and light responses (CIACADIANLELHC and PIF3). Both motif prediction programs identified motifs corresponding to the known CREs AG, AGL3, Athb-9, O2 and RAV1 (Table 4).

**Table 4:** **Known *cis*-regulatory elements (CREs) in the promoter regions of *NP-like *genes within *Populus trichocarpa *identified by the motif prediction programs MEME and MotifClick (MC), CRE database and the transcription factors and/or functions**

**CRE name**	**Prediction Program**	**CRE database**	**Transcription factor/function**
AG	MC	TRANSFAC	AGAMOUS, expressed in flowers
	MEME		
AGL3	MEME	TRANSFAC	AGAMOUS-like (AGL) 3, expressed in vegetative and floral above ground tissue
	MC		
ANT	MEME	TRANSFAC	ANT (a member of AP2/EREBP TFs)
ARR10	MC	JASPER	ARR10, involved in 2 component regulation and possibly cytokinin signaling
Athb-1	MC	TRANSFAC	AtHB-1, involved in cell differentiation in leaves, expressed in leaves
Athb-5	MC	TRANSFAC	AtHB-5, expressed in vegetative tissues, preferentially in leaf tissues. Function in mature vegetative tissues.
Athb-9	MC	TRANSFAC	AtHB-9, possibly involved in dorsiventral patterning of lateral organs (leaves).
	MEME		
bZIP910	MC	TRANSFAC	BZIP transcript factor from snapdragon.
C8GCARGAT	MC	PLACE	AGL15, possibly involved in gibberellin metabolic signaling.
CARGCW8GAT	MC	PLACE	AGL15, possibly involved in gibberellin metabolic signaling.
CDC5	MEME	TRANSFAC	AtCDC5, required for function of shoot apical meristem. Silencing accelerates cell death in leaves. Possibly involved in cell cycle regulation.
CIACADIANLELHC	MC	PLACE	Region necessary for circadian expression of light harvesting complex genes
ERELEE4	MC	PLACE	Ethylene responsive element, senescence-related expression
GMHDLGMVSPB	MC	PLACE	GmHdl56/GmHd157, found in the promoter vegetative storage protein conferring function vacuolar glycoprotein acid phosphatase in soybean
HMG-IY	MEME	JASPER	Binding regions for proteins similar to histone H1/H5 family
MYB.ph3	MC	JASPER	MYB.ph3, petal epidermis-specific, possibly GA regulated and may bind chromatin
		TRANSFAC	
O2	MC	TRANSFAC	Opaque-2, found in Maize endosperm
	MEME		
PIF3	MEME	TRANSFAC	PIF3, present in many light-regulated promoters, including PhyB
RAV1	MC	TRANSFAC	RAV1, may be negative regulator of plant development, down regulated by brassinosteroids. Touch-, drought-, salt- cold-, bacteria-induced. Positively regulates leaf senescence. Ethylene mediated signaling.
	MEME		
RYREPEATVFLEB4	MEME	PLACE	FUS3/TRAB1, ABA and auxin responsive, found in seed proteins
SQUA	MC	JASPER	SQUA, required for inflorescence development
UPRE2AT	MEME	PLACE	Found in promoters of many genes associated with ER stress
UPRMOTIFIAT	MEME	PLACE	

**Figure 6:**
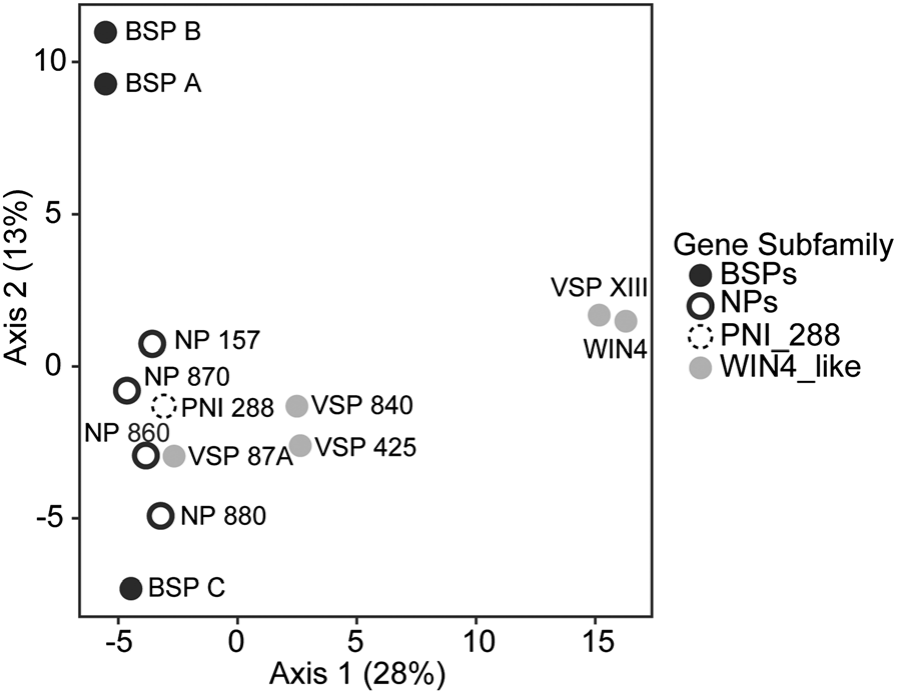
**Principal component analysis (PCA) of *NP-like cis*-regulatory element motifs in *Populus trichocarpa*.** PCA based upon the abundance of *cis*-regulatory element motifs within promoter regions of *NP-like* genes in *Populus trichocarpa*. The percentage of variation explained by each axis is given in parentheses next along each axis.

The results of the principal component analysis based on the number of predicted motifs within each gene were generally consistent with the phylogenetic relationships. Along PCA Axis 1, which explains 28% of the variance in the data, motifs in the promoter regions of *VSP XIII* and *WIN4* genes are primarily separated from the *BSP* and *NP* genes (Figure 6). PCA Axis 2, which explains 13% of the variance in the data, primarily separates the motifs in the promoter regions of the *BSP* genes from the other *NP-like* genes. However, along axis two the motifs in the promoter regions of *BSP A* and *BSP B* are found close together compared to motifs of the promoter of *BSP C* which is isolated at the negative end of the axis. Based on the *λ*_*k*_ approach described above to assessing the number of non-trivial components, we found that axes 1 and 2 had eigenvalues significantly greater (*P* < 0.05) than those created under the randomizations.

### Phylogenetic distribution in the plant kingdom

**Figure 7:**
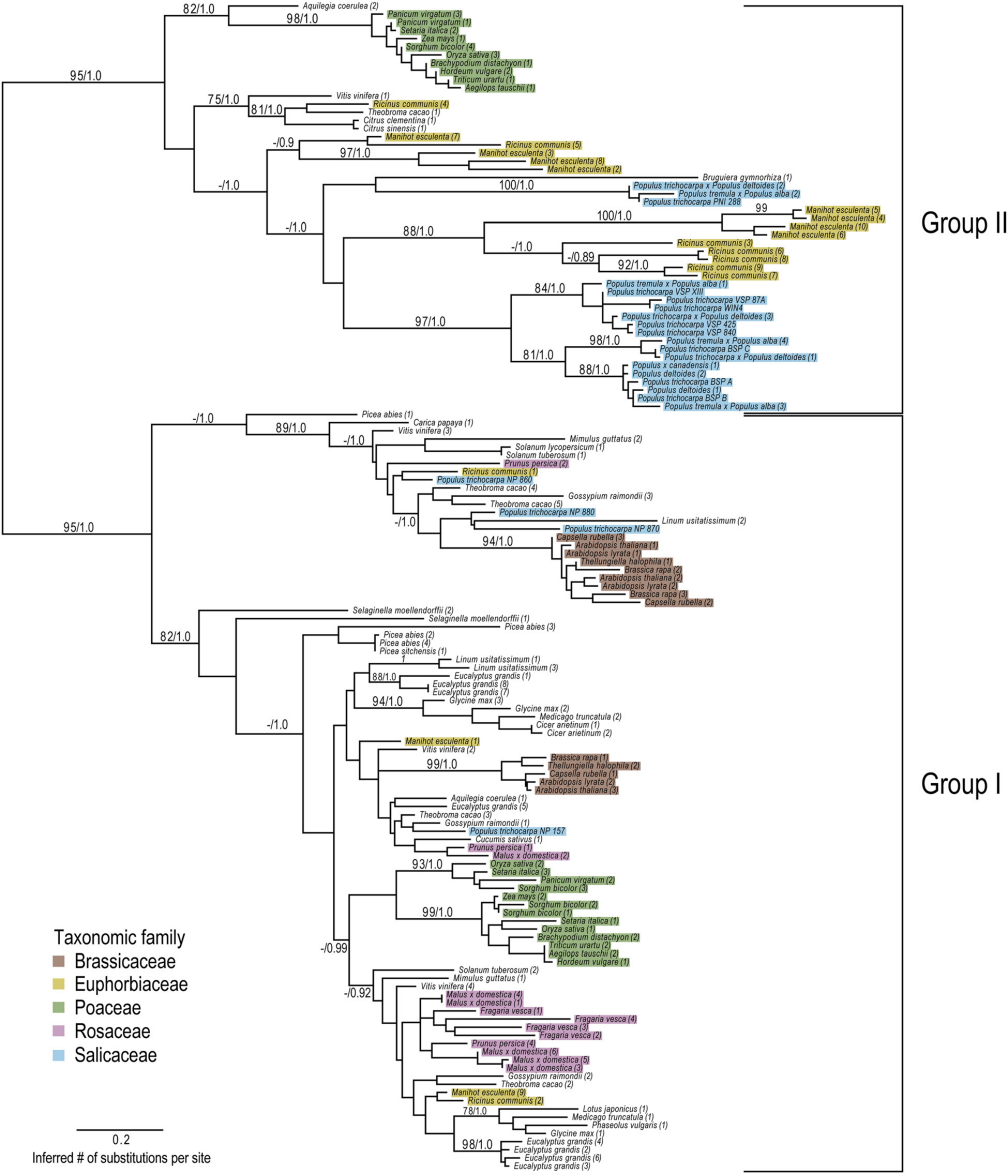
**Phylogenetic analyses of NP-like proteins in the plant kingdom.** Phylogenetic relationships were constructed using Bayesian and maximum-likelihood methods. Numbers at branches indicate posterior probabilities and bootstrap percentages based on 1000 replicates, respectively. Numbers in parentheses correspond to Phytozome or NCBI sequence identifiers, which can be found in the Additional file 7: Table S4. The five predominant taxonomic families are indicated by the highlighted colors.

To investigate the diversity of plant NP-like proteins, we retrieved 142 complete and non-redundant amino acid sequences across the plant kingdom (Additional file 2: Table S5) with NP sequence homology based on BLASTP search. Phylogenetic analyses based on alignments of the predicted NP-like region for 38 genera revealed 2 general groups of NP-like proteins (Figure 7). Group I and II have strong support, with posterior probabilities of 1.0 as well as bootstrap support of 95%. Group I includes NP-like proteins from all represented genera having complete genome sequences with subclasses that generally cluster according to Rosids and monocot lineages [44,45]. Group I also includes *Populus* proteins that are not known to be involved in storage (NP-like subfamily members). Phylogenetic analyses that include a bacterial outgroup can be found in Additional file 3: Figure S1. The addition of an outgroup does not change the topology of the plant NP groups.

Group II is mainly composed of proteins from *Populus*, *Manihot esculenta* and *Ricinus communis.* There is a smaller cluster of proteins from monocot genera and two citrus proteins. The *Populus* proteins in Group II include the BSPs, WIN4-like proteins and PNI 288 (Figure 7).

## Discussion

### Protein phylogeny and chromosomal distribution of *NP-like* gene family in *Populus*

Previous studies identified three *BSP* genes, three *WIN4-like* genes and *PNI 288*[15]. In this study we examined the *Populus* genome sequence and identified an additional 6 genes to expand this gene family to 13 members. Based on our phylogenetic analyses we propose this gene family be designated the *NP-like* gene family with three subfamilies that include the *BSP* subfamily (three gene members), the *WIN4-like* subfamily (five gene members) and the *NP-like* subfamily (four gene members). In addition, *PNI 288* also is a member of the NP-like gene family but does not cluster with any of the three subfamilies.

The protein phylogeny of the evolutionary relationships among NP-like proteins across the plant kingdom and the chromosomal distribution indicates that the *NP-like* gene family in *Populus* expanded through WGD and TD events. This is consistent with previous studies investigating the evolutionary history of the *Populus* genome [46-48]. A WGD event occurred in *Populus* approximately 65 mya (millions years ago) or earlier, followed by genome wide reorganization that resulted in, among other changes, paralogous sets of chromosomes of which XIII and XIX are a pair (where *PNI 288* and the *BSP*/*WIN4-like* subfamilies reside, respectively) [46-48]. Chromosomes VI and VIII (where *NP 157* and the *NP-like* subfamily reside, respectively) are not a paralogous set [46]. The members of each gene subfamily are located within a 100 Kb region and are likely the result of TD (Figure 2). This proximity of the genes to each other is generally a good indication of TD [49,50]. The type of duplication event (i.e. TD or WGD) has implications on gene function: genes families in *Populus* that have expanded through TD are enriched for functions involving defense responses, apoptosis and protein kinases [49].

### *In silico* promoter analyses of the *NP-like* gene family in *Populus*

Promoter analyses are an important component of investigating the functional evolution of genes particularly in cases where genes are created by TD. This is because TD mainly occurs through unequal recombination, which can result in subfunctionalization if regulatory regions are not also duplicated [51]. These analyses also provide information regarding possible regulation and tissue-specific expression—crucial for future investigations to determine function.

Despite the potential for informative conclusions to be gained from *in silico* promoter analyses, motif discovery is difficult from a bioinformatics perspective [52]. Mapping CREs in promoter regions using only databases of known CREs results in a high rate of false positives and does not prove that these regions are TF binding sites. To increase confidence, we utilized 2 motif prediction programs (MEME and MotifClick) to identify over-represented 8 bp motifs in the 2 kb promoter regions of *NP-like* genes in *Populus*. Known CREs were identified from the resulting motifs and mapped (Table 4, Figure 5). Both programs predicted motifs that correspond to CREs associated with gene activation by the TFs AG, AGL3, Athb-9, O2 and RAV1. These CREs are involved in a wide range of developmental and metabolic processes. AG has been shown to bind the AGAMOUS TF that functions in temporal development of floral stem cells [53] while the related TF AGL3 is likely involved in development of above-ground organs and binds to regions identified as the AGL3 CRE [54]. Athb-9 appears to regulate patterning of leaves while O2 or Opaque-2 is a TF responsible for expression of a prominent storage protein in *Zea mays* and contribute to endosperm metabolism [55,56]. Lastly, RAV1 is a TF that promotes and possibly regulates leaf senescence [57]. Overall, we identified CREs that have been shown to be involved in light, stress and defense and hormone responses. The presence of these potential CRE in the promoters of the *Poplar NP-like* genes provides possible clues related to the regulation of these genes. The potential regulation of *NP-like* genes in *Populus* by hormone signaling pathways would be particularly interesting to pursue.

The principal component analysis of predicted promoter motifs is consistent with the similarity tree expression data (Figure 1A) and phylogenetic analyses (Figure 6). This is not unexpected since gene expression is regulated by TFs and their interaction with CREs in promoter regions. The distances between *BSP A*, *BSP B* and *BSP C*, which represent differences in the motifs in their promoter regions, could result from unequal recombination combined with diversifying selection (Figure 6, 1A) [51]. This may also explain the distances between the motifs in the promoter regions of *WIN4-like* genes (Figure 6, 1A).

### Expression divergence and natural selection analyses

Our evaluation of selection pressure indicates diversifying selection at the initial duplication event and more recent divergent selection of *BSP* and *WIN4-like* subfamily members. This succession of duplication events is consistent with phylogenetic studies in plants (e.g. [58-60]). *Populus* specific duplication events have also been evaluated [46,48,60]. In *Populus* there are reports that correlate tandemly duplicated genes to the expansion of plant defense and stress gene families, consistent with the gene dosage model [49,50,61]. Strong patterns between opposing functional gene groups retained following either WGD or TD events have been observed in *P. trichocarpa*[49,58]. This fits well with observations of BSPs and VSPs accumulating following wounding and drought stress [22,23].

Our results show that *BSP* and *WIN4-like* subfamilies gene expression patterns evolved relatively recently and members of these subfamilies are also under selection pressure; this suggests they are undergoing subfunctionalization or diversification of multifunctional genes. This is supported by the expression data showing differential co-expression among the *BSP* and *WIN4-like* gene subfamilies (Figure 1B). Perhaps the data reflect amplification, buffering or “near” subfunctionalization, all of which can facilitate functional redundancy that enables organisms to respond to a greater range of cellular, environmental and/or genetic perturbations [62,63]. This positive dosage model is particularly applicable to genes involved in stress and environmental responses [29,30,37]. Such a strategy would functionally promote optimum nutrient cycling and storage pathways [24]. The amplification model describes the potential functional evolution and fixation of genes from unstable (i.e. tandem) duplications [31]. The high degree of nucleic acid similarity between *BSP* genes (*BSP A* vs. *BSP B*: 98.2%; *BSPA* + *B* vs. *BSP C* ~94%) also supports the amplification model since sequence divergence is loosely correlated with expression divergence [49].

We qualify our predictions of the functional gene fates, acknowledging that classification is restricted to the current gene duplication models, which are limited by a poor understanding of the role of population size, selection pressure and fixation preceding and following duplication [26,64,65]. Research is trending toward developing a more general model of adaptive maintenance duplication [64-66]. Without such information it is particularly difficult to distinguish the subfunctionalization models and the diversification of a multifunctional genes [66]. Another complexity involves multiple types of functionalization occurring over the course of evolution [67].

### *NP-like* gene family expansion order within *Populus trichocarpa*

**Figure 8:**
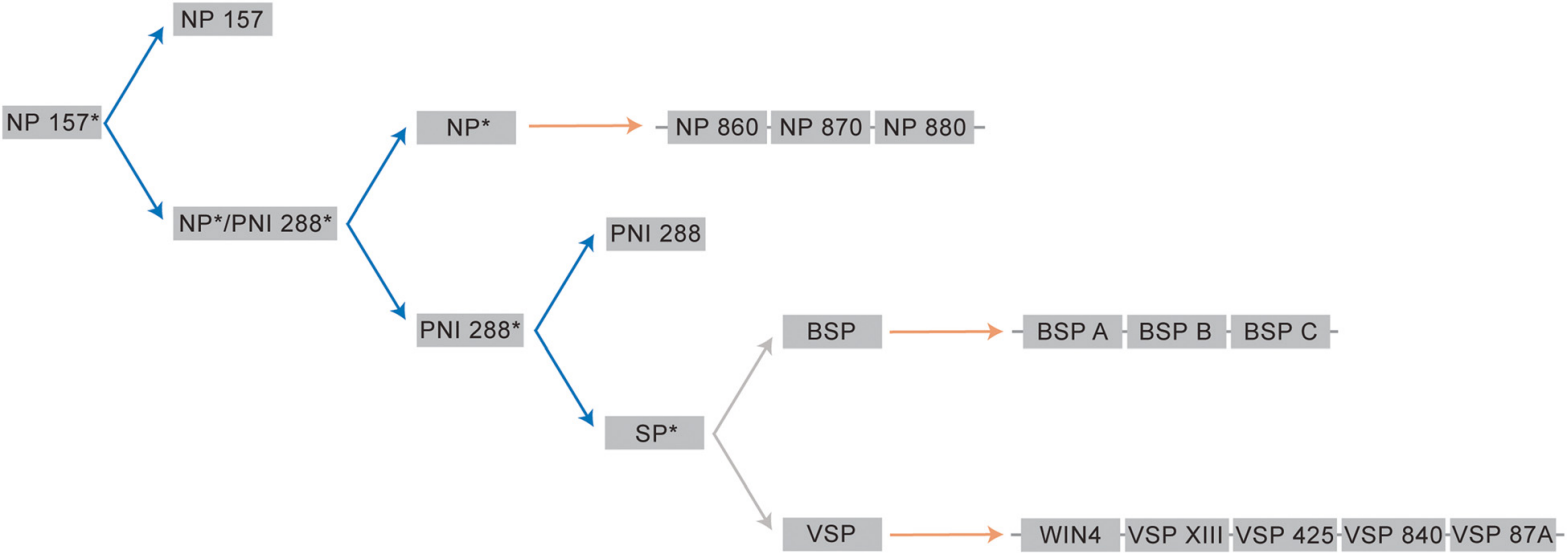
**Hypothetical origin of the *NP-like *gene family in *Populus trichocarpa*.** Blue arrows represent whole genome duplication events and orange arrows represent tandem duplication events. Asterisks denote ancestral genes with unknown sequence similarity to present-day genes.

From the results presented here, we propose a hypothetical origin of *NP-like* genes within *Populus trichocarpa,* depicted in Figure 8. The NP-like parent protein was retained following an ancient WGD event after which the NP-like parent protein and the duplicate became the progenitors of Group I and II NP-like proteins, respectively. These genes could have been retained if they had a function involvement in signaling networks or pathways, the dosage balance model. The progenitor of Group II NP-like proteins may have undergone a functionalization process that conferred fitness or became advantageous following a change (i.e. environmental) followed by stabilizing selection pressure. Another WGD event occurred, specific to the *Populus* lineage and was followed by tandem duplications where upon the amplification of this function would result in immediate stabilizing selection pressure, the positive dosage model.

Our selection and phylogenetic analyses suggest *NP 157* is the likely progenitor gene. Further, the expression patterns of *NP 157* were distinctly different from those of the *BSP* and *WIN4-like* gene subfamilies. It seems unlikely that resources would be directed toward transcription of high levels of *NP 157* transcripts, and presumably the translation of proteins, of a nonfunctional or pseudogene in senescing leaves. Two explanations are that NP 157 is scavenging nucleosides prior to senescence or has retained regulatory elements similar to those of other genes involved in purine salvage and degradation. Increased expression of other genes involved in purine salvage and degradation has been observed under dark-induced senescence conditions [14]. Additionally, knocking-down the enzyme considered to be the key bottleneck in purine degradation, xanthine dehydrogenase, resulted in reduced growth, early senescence and infertility [12,68]. Yet another explanation could be that NP 157 acts as transient storage protein or is otherwise involved in nutrient signaling. At the very least, *NP 157* as well as *BSP* and *WIN4-like* genes may be responding to changes in nutrient signals induced by SD photoperiod and/or the onset of senescence. This seems plausible when compared to *NP* expression in other systems. In the bacteria *Bacillus subtilis*, *PNP* transcription is induced by nucleosides in growth media and repressed by glucose, pointing to modulation of *PNP* by carbon and energy availability [69,70]. In humans, *PNP*s are up-regulated in diseased and cancerous tissues where metabolic shifts occur [4-6]. While more research is needed to conclusively define the function of NP 157 we posit that NP-like proteins may still be involved in nutrient signaling in plants.

### Phylogenetic distribution across the plant kingdom

Our data suggest that NP-like proteins were conserved in all higher plants examined with complete genomes (Figure 7). Interestingly, NP-like proteins were not identified in *Physcomitrella* and green algae genomes. These taxa could have lost NP-like genes. In bacteria, there are multiple phyla that do not have PNPs or related uridine phosphorylases and it is hypothesized that this may have occurred due to different resource requirements among phyla in bacteria [71]. The non-vascular plant *Selaginella moellendorffii* has two NP-like representatives that cluster at the base of Group I NP-like proteins suggesting that these proteins are the most ancestral (Figure 7). The inclusion of a bacterial outgroup supports the hypothesis that NP-like proteins are monophyletic and evolved from NPs (Additional file 3: Figure S1).

The phylogeny of NP-like proteins across the plant kingdom reveals that the *P. trichocarpa* subfamily designations are consistent with those found in the tree of the 13 NP-like proteins in *Populus* (Figures 1 and 5). The BSP and WIN4-like subfamilies and PNI 288 cluster within Group II and the NP-like subfamily belong to the Group I. If NP-like proteins functioned as storage proteins in all plants, we would expect that NP-like proteins from other woody perennial genera would cluster near *Populus* storage proteins yet *Prunus*, *Picea* and *Malus* NP-like proteins do not cluster near *Populus* storage proteins. Instead, proteins from these genera cluster in Group I near proteins from the NP-like subfamily members in *Populus*. This suggests that NP-like proteins are not storage proteins throughout the plant kingdom.

In addition to the conclusions based on our phylogenetic analyses, the finding that NP-like proteins are not general storage proteins is consistent with previous reports on seasonal storage proteins in other woody plant genera. For example, *Prunus persica* and *Picea sitchensis* are both woody perennials. In the bark of *Prunus persica*, the predominant storage proteins are a 60 kDa dehydrin, a 19 kDa allergen-related protein and a 16 kDa protein with no known homology [72]. In *Picea glauca x engelmannii complex*, the predominant storage proteins were 20 and 27 kDa, loosely suggesting that 32 kDa BSPs are not the dominant storage proteins in *Picea*[73].

Our results point to an expansion of the *NP-like* gene family in a common ancestor of the order *Malpighiales* of which *Populus, Manihot, Ricinus* and *Linum* are representatives [44,74]. While proteins from *Populus, Manihot and Ricinus* often clustered together in both Groups, *Linum*, has only three NP-like proteins that cluster in Group I. We propose that *Linum* lost Group II NP-like proteins. The recent assembly of the *Linum* genome revealed that *Linum* has experienced a recent WGD followed by the loss of one or more chromosomes [75]. The many proteins found in *Manihot* and *Ricinus* that cluster near the *Populus* storage proteins indicates a storage function in these genera.

## Conclusions

Our investigation into the functional significance and evolutionary history of NP-like proteins and genes within *Populus* and across the plant kingdom illustrates the importance of both microevolutionary (e.g., natural selection) and macroevolutionary (e.g., genome duplication and tandem duplications) forces in shaping patterns of diversity within this protein family. Of particular note is that we found evidence that hitherto uncharacterized *NP-like* genes might serve a functional role within *Populus* based on expression data; the functional significance of such proteins is unknown. Further, analyses of promoter regions that showed the presence of motifs associated with light responses, phytohormone responses, stress and tissue-specificity that also support a functional role for NP-like proteins. We also found significant evidence for episodic diversifying selection acting on the NP-like proteins within *Populus* and that changes in gene expression levels had occurred relatively recently in the evolution of this gene family. Consistent with the other findings, our inferred phylogeny implicates both historical genome duplication events and more recent taxon specific independent duplication events as mechanism that gave rise to the extant diversity of NP-like proteins within *Populus.* In conclusion, our results are the first to examine NP-like proteins in plants and we present the possibility that they may have functional significance within plants.

## Methods

### Plant material

*Populus trichocarpa* (Torr. and Gray) genotype ‘Nisqually-1’ cuttings were grown in 6 L pots in controlled environmental chambers at 18°C with a PAR range of 310–470 *μ*mol m^–2^ s^–1^. Plants were fertilized one week after transplanting with 5 g of controlled release fertilizer (18-3-3) (Nutricote, Florikan, Sarasota, FL, USA). Tissue samples were collected after 8 weeks under LD conditions (16 h light/8 h dark) and after 3, 6, 8 and 12 weeks under SD conditions (8 h light/16 h dark). The temperature was lowered to 10°C day/4°C night for the last 4 weeks of SD (i.e. from 8 to 12 weeks SD). Shoot tips or apical buds, young leaves (leaf plastochron index (LPI) 3), mature leaves (LPI 8) and bark (between LPI 8–9) were harvested and frozen in liquid N_2_ and then stored at -80°C until RNA extraction. One replicate was composed of pooled tissues from three plants. Two replicates were used for gene expression analysis.

### Identification and phylogenetic analysis of NP-like genes in the *Populus* genome

To identify NP-like proteins in the *Populus* genome a BLASTP search against the *Populus trichocarpa* genome v2.2 (http://www.phytozome.net) was performed using default parameters (expect (E) threshold = -1, BLOSUM62 comparison matrix) with the *P. trichocarpa BSP A* (locus name: POPTR_0013s10380) as the query sequence. NP-like protein sequences meeting these criteria were then retrieved from the Phytozome database and used for phylogenetic analyses.

### QPCR primer design and validation

Primers for the NP-like gene family and reference genes were designed using MacVector v10 (MacVector Inc., Cary, NC, USA). All primers were synthesized by Invitrogen (Carlsbad, CA, USA). Optimum annealing temperatures were determined by a temperature gradient and amplification efficiencies calculated from a five-point calibration curve of ten-fold serial dilutions. To confirm a single amplification product, melt curves were performed for all qPCR reactions.

### RNA extraction, cDNA synthesis, qPCR detection and statistical analyses

Total RNA was purified using RNeasy plant mini kits and the automated QIAcube (Qiagen, Valencia, CA, USA). Tissues were ground in liquid nitrogen and added to RLT extraction buffer containing 1% polyvinylpyrrolidone, and 1% beta-mercaptoethanol. Samples were vortexed and 0.4 volumes of 5 M potassium acetate, pH 6.5 was added to the sample, mixed and incubated on ice for 15 min. Samples were then centrifuged at 15,000 g for 15 min at 4°C. The supernatant was transferred to the QIAcube for RNA extraction with on-column DNase I digestion (Qiagen, Valencia, CA, USA). Microfluidic analyses using the Experion^TM^ automated electrophoresis system and RNA StdSens Chips were used to determined RNA quality and quantity (Bio-Rad, Hercules, CA, USA). First strand cDNA was synthesized in triplicate reactions from 1 μg of total RNA using the RevertAid First Strand cDNA Synthesis Kit (Thermo Scientific Inc., Rockford, IL, USA) and oligo dT primers. cDNA was pooled and used as template for qPCR detection. Amplification reactions were performed with the iQ5 Real-Time Detection System (Bio-Rad, Hercules, CA, USA) using Maxima SYBR green qPCR master mix (Thermo Scientific Inc., Rockford, IL, USA). Amplification reactions consisted of 10 min at 95°C followed by 40 cycles of 15 sec at 95°C and 1 min at the optimum annealing temperature. Stable reference genes were determined for each tissue type by geNorm^PLUS^ in qbase^PLUS^ version 3 (http://www.qbaseplus.com). (Additional file 4: Table S1) and relative expression analyses were performed with qbase^PLUS^ (Additional file 5: Table S2). Relative expression data are scaled to the maximum relative expression units of each tissue.

We also performed a principal component analysis based on differences in expression patterns within each tissue type across the treatments for each of the 13 genes. The analysis was performed using the ADE4 package [76] within R [77].

### *In silico* promoter sequence analyses of the *NP-like* gene family in *Populus*

Promoter sequences 2 kb upstream from the transcription start site (ATG codons) of each *Populus NP-like* genes were retrieved from the *Populus trichocarpa* genome v2.2 in Phytozome (http://www.phytozome.net/). *In silico* analyses of these regions were performed using the prediction programs MEME (http://meme.nbcr.net) and MotifClick (http://motifclick.uncc.edu/) to determine overrepresented 8 bp motifs [41,42]. MEME uses an expected-maximization strategy to detect overrepresented motifs whereas MotifClick employs a graph-based algorithm that determines motifs by identifying complete subgraphs also known as cliques [41,42]. MEME was performed with the following settings: any number of repetitions for a single motif distributed among the sequences, the minimum and maximum motif width of 8 bp and the maximum number of motifs to find was 100. Settings for MotifClick analyses were performed with motif length set to 8 bp, 100 maximum motif number, more degenerate sites and sum of square distance between the binding site and background sequence run at 0.06 and 0.1. The output from runs at SSD 0.06 and 0.1 were 93% similar, with output from SSD 0.06 being chosen for further comparison (Additional file 6: Table S3). The resulting 8 bp motifs were compared against the known CRE sequences in these regions determined by PlantPAN (Plant Promoter Analysis Navigator) [43]. PlantPAN collected known TF binding motifs from PLACE, TRANSFAC, AGRIS and Jaspar [43] (see Additional file 7: Table S4 for output from PlantPAN). Motif lengths of 8 bp were chosen for three reasons: TFs commonly bind to DNA regions that are between 8–10 and 16–20 bp in length, the length is suitable for prediction programs and cross-referencing with output of PlantPAN [52]. Smaller regions can result in an increased number of false-positives [52].

We conducted a principal components analysis to elucidate the relationships among the proteins based on the abundance of motifs predicted by both programs. The input for the analysis, which were conducted using the ADE-4 package [76] within R [77], was a matrix of motif counts per gene. To identify non-trivial components produced by PCA, we performed an analysis method [78] of 10000 randomized matrices of the dataset in R [77].

### Protein sequence identification and motif prediction of NP-like region for phylogeny

For constructing the evolutionary relationships among NP-like proteins across the plant kingdom, we retrieved amino acid sequences from the Phytozome database (http://www.phytozome.net) using a BLASTP search with default parameters and the *P. trichocarpa BSP A* (locus name: POPTR_0013s10380) protein sequence for the query. For species absent in Phytozome, a BLASTP search with default parameters was performed at NCBI using the “non-redundant protein sequence” database (http://www.ncbi.nlm.nih.gov/). Sequences for *Malus* were retrieved from the Rosaceae Database (http://www.rosaceae.org/) and sequences for *Picea abies* were retrieved from ConGenIE (http://congenie.org/). For all searches, only hits below an E-value of 10^-4^ were used. Sequences were manually inspected for annotation errors and duplicate sequences were removed. A total of 142 sequences were used in the analysis and 3 bacterial sequences that serve as an outgroup (Additional file 2: Table S5). To confirm that all plant sequences could be classified as NPs, a batch CD-search of the Conserved Domain Database (CDD) using all 142 plant sequences was performed with default setting and the E-value set to 0.01 with all sequences identified as members of the PNP_UDP_1 superfamily (Pfam:01048) [79]. The conserved region was identified with the Gapped Local Alignment of Motifs (GLAM2) program, version 4.8.1, for all sequences with default parameters and 8,000 iterations (http://meme.nbcr.net/meme/cgi-bin/glam2.cgi) [80]. Predicted motifs were cross-referenced with GLAM2SCAN using the NCBI non-redundant protein database with closest matching motifs in proteins within PNP_UDP_1 superfamily (Pfam:01048). To validate GLAM2 predicted motifs, 57 non-plant (bacterial) sequences from the NP family (COG0775, superfamily cl00303) were retrieved from CDD (http://www.ncbi.nlm.nih.gov/Structure/cdd/cdd.shtml) and motifs were predicted for this set of sequences by GLAM2. We then constructed alignments of the predicted motifs from each sequence set (plant and non-plant) with MUSCLE set to default settings to validate the NP-like region in plants [81] (Additional file 8: Figure S2). The validated NP-like motif in plants was used to remove regions outside the NP-like motif sequence and the resulting sequences were used for phylogenetic tree construction.

The coordinates of syntenic regions within the *Populus trichocarpa* genome v2.2 represented in Figure 2 can be found at http://ftp.jgi-psf.org/pub/compgen/phytozome/v5.0/ Ptrichocarpa/miscelaneous/synteny/allPoplarSegments.seg.

### Phylogenetic analyses and protein family evolution

Phylogenetic hypotheses were constructed for each alignment using both a Bayesian (MrBayes v3.0; [82]) and maximum-likelihood based method (Genetic Algorithm for Rapid Likelihood Inference; GARLI; [83]). We used the WAG + I + G amino acid substitution model, determined by ProtTest [84], for all analyses (including the phylogeny of NP-like proteins in *Populus* with full length amino acid sequences). We present the best topology based on 1000 replicate GARLI analyses on the observed dataset and assessed the statistical support for topological relationships from 1000 bootstrap replicates. All GARLI analyses were performed using the computing resources associated with the LATTICE project [85]. For the Bayesian analyses, we used the default settings (two concurrently running independent analyses of four chains, three of which were heated) but set the amino acid model to mixed. We used a threshold of 0.01 for the standard deviation of the split frequencies as a measure of sufficient convergence and mixing. Analyses of the smaller 13 protein *Poplulus* dataset and the larger 142 protein dataset were run for 10^6^ and 10^7^ generations, respectively. In both analyses, 25% of the generations served as burn-in. Multiple sequence alignment and phylogenetic tree are available from TreeBASE (http://purl.org/phylo/treebase/phylows/study/TB2:S14494; [86]). Phylogenetic analyses that include a bacterial ourgroup can be found in Additional file 3: Figure S1.

### Tests of natural selection and gene expression evolution

We tested for recombination in the 13-gene *Populus* dataset, which can adversely affect analyses of natural selection, using the Genetic Algorithm for Recombination Detection (GARD) method [87] implemented in the package HyPhy [88]. The results from GARD did not identify any statistically significant evidence for recombination breakpoints. Thus, we used the Branch-site REL (random effects likelihood) method [87] implemented in the package HyPhy to determine whether any of the gene duplicates within the 13 gene *Populus* dataset showed evidence for being under diversifying selection. We chose this method of detecting selection since our primary interest was to determine which gene duplicates, rather than sites, are under selection. This does not make assumptions as to what branches may be under selection, which can increase the incidence of false positives [87]. In all analyses, the codon sequences were used.

We performed two different tests to assess the evolutionary history of gene expression patterns within each of the four tissue types. First, we estimated the parameter λ, under which 0 represents a complete lack of phylogenetic signal and 1 indicates a strong phylogenetic signal (i.e., similarity due to shared ancestry). Second, we tested whether the evolution of gene expression levels has increased, decreased, or been constant over time by estimating the parameter δ; δ < 1 indicates that the evolution of differences in gene expression patterns occurred early in gene divergence and δ > 1 signifies that evolutionary differences occurred relatively recently. To assess the statistical fit of λ and δ models, we performed a likelihood ratio test (LRT) with respect to the results under a Brownian motion model of character evolution. All analyses were conducted using the GEIGER package [89] within R [77].

## Competing interests

The authors declare that there are no competing financial interests.

## Authors’ contributions

EP devised experimental setup, performed all work for expression evaluation and promoter analyses and wrote the manuscript. EP and JP conducted analyses. GC offered directional support. JP and GC participated in manuscript editing. All authors have approved the manuscript.

## Supplementary Material

Additional file 1: Table S6 Known *cis-*regulatory elements that correspond to matching predicted motifs. Includes *cis-*regulatory element (CRE) name, sequence, CRE database and additional functional information. Click here for file

Additional file 2: Table S5 Phylogenetic tree identifiers. Identifiers from Figure 7 corresponding to the Phytozome locus or NCBI sequence identifier. Click here for file

Additional file 3: Figure S1 Phylogenetic analyses of NP-like proteins in the plant kingdom with bacterial outgroup. Phylogenetic relationships were constructed using Bayesian and maximum-likelihood methods. Numbers at branches indicate posterior probabilities and bootstrap percentages based on 1000 replicates, respectively. Numbers in parentheses correspond to Phytozome or NCBI sequence identifiers, which can be found in the Additional file 7: Table S4. The five predominant taxonomic families are indicated by the highlighted colors. Click here for file

Additional file 4: Table S1 QPCR reference gene information. Includes qPCR reference gene symbol, gene name, Phytozome locus name, primer sequences, size of amplification product, annealing temperature, PCR efficiency and the tissue type that the reference gene was used for normalization calculations. Click here for file

Additional file 5: Table S2 Un-scaled relative expression of *NP-like* gene family in *Populus*. Click here for file

Additional file 6: Table S3 Promoter motifs predicted by MEME and MotifClick. Eight bp promoter motifs predicted by MEME (left) and MotifClick (right). Click here for file

Additional file 7: Table S4 Output from PlantPAN. Includes the *cis*-regulatory element sequence, *NP-like* gene following the promoter region, regulatory element name, location within the promoter region, database element was retrieved from and functional category. Click here for file

Additional file 8: Figure S2 Alignment of motifs predicted by MEME for bacterial and plant nucleoside phosphorylase (NP) proteins. Bacterial amino acid sequences were retrieved from the Conserved Domain Database (CDD, http://www.ncbi.nlm.nih.gov/Structure/cdd/cdd.shtml) from the NP family (COG0775) and plant amino sequences were retrieved from Phytozome (http://www.phytozome.net). Click here for file
