# Supplementary material for: Elucidating the evolutionary history and expression patterns of nucleoside phosphorylase paralogs (vegetative storage proteins) in Populus and the plant kingdom
Source: BMC Plant Biol. 2013 Aug 19;13:118. doi: 10.1186/1471-2229-13-118 (PMC3751785; doi:10.1186/1471-2229-13-118)
Supplement: Additional file 3: Figure S1 — Phylogenetic analyses of NP-like proteins in the plant kingdom with bacterial outgroup. Phylogenetic relationships were constructed using Bayesian and maximum-likelihood methods. Numbers at branches indicate posterior probabilities and bootstrap percentages based on 1000 replicates, respectively. Numbers in parentheses correspond to Phytozome or NCBI sequence identifiers, which can be found in the Additional file 7: Table S4. The five predominant taxonomic families are indicated by the highlighted colors. [file 1471-2229-13-118-S3.pdf]

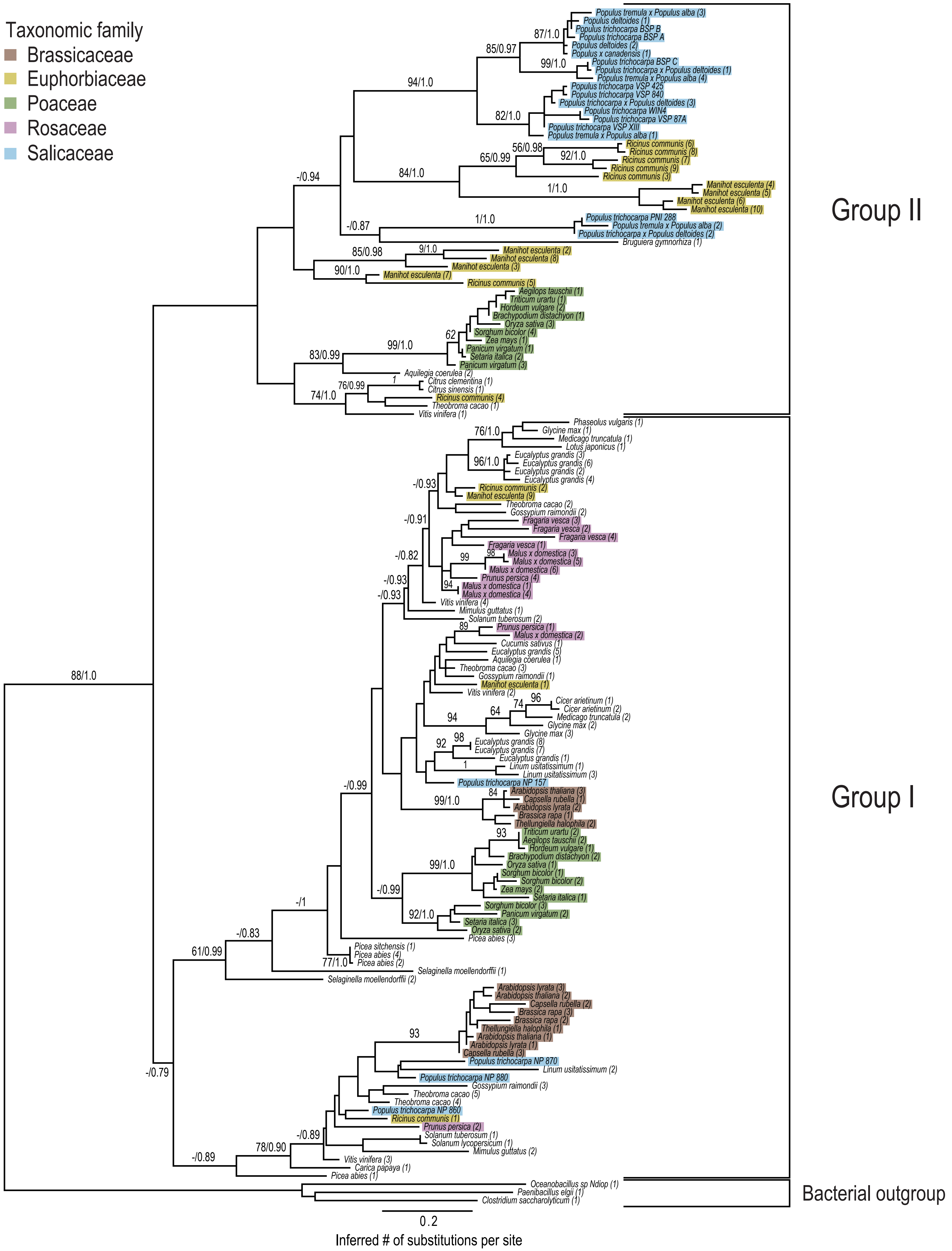
Taxonomic family
Brassicaceae
Euphorbiaceae
Poaceae
Rosaceae
Salicaceae
Populus tremula x Populus alba (3)
Populus deltoides (1)
Populus trichocarpa BSP B
Populus trichocarpa BSP A
Populus deltoides (2)
Populus x canadensis (1)
Populus trichocarpa BSP C
Populus trichocarpa x Populus deltoides (1)
Populus tremula x Populus alba (4)
Populus trichocarpa VSP 425
Populus trichocarpa VSP 840
Populus trichocarpa x Populus deltoides (3)
Populus trichocarpa WIN4
Populus trichocarpa VSP 87A
Populus trichocarpa VSP XIII
Populus tremula x Populus alba (1)
Ricinus communis (6)
Ricinus communis (8)
Ricinus communis (7)
Ricinus communis (9)
Ricinus communis (3)
Manihot esculenta (4)
Manihot esculenta (5)
Manihot esculenta (6)
Manihot esculenta (10)
Populus trichocarpa PNI 288
Populus tremula x Populus alba (2)
Populus trichocarpa x Populus deltoides (2)
Bruguiera gymnorhiza (1)
Manihot esculenta (2)
Manihot esculenta (8)
Manihot esculenta (3)
Manihot esculenta (7)
Ricinus communis (5)
Aegilops tauschii (1)
Triticum urartu (1)
Hordeum vulgare (2)
Brachypodium distachyon (1)
Oryza sativa (3)
Sorghum bicolor (4)
Zea mays (1)
Panicum virgatum (1)
Setaria italica (2)
Panicum virgatum (3)
Aquilegia coerulea (2)
Citrus clementina (1)
Citrus sinensis (1)
Ricinus communis (4)
Theobroma cacao (1)
Vitis vinifera (1)
87/1.0
85/0.97
99/1.0
94/1.0
82/1.0
56/0.98
65/0.99
92/1.0
84/1.0
1/1.0
-/0.94
1/1.0
-/0.87
9/1.0
85/0.98
90/1.0
62
99/1.0
83/0.99
1
76/0.99
74/1.0
Group II
Phaseolus vulgaris (1)
Glycine max (1)
Medicago truncatula (1)
Lotus japonicus (1)
Eucalyptus grandis (3)
Eucalyptus grandis (6)
Eucalyptus grandis (2)
Eucalyptus grandis (4)
Ricinus communis (2)
Manihot esculenta (9)
Theobroma cacao (2)
Gossypium raimondii (2)
Fragaria vesca (3)
Fragaria vesca (2)
Fragaria vesca (4)
Fragaria vesca (1)
Malus x domestica (3)
Malus x domestica (5)
Malus x domestica (6)
Prunus persica (4)
Malus x domestica (1)
Malus x domestica (4)
Vitis vinifera (4)
Mimulus guttatus (1)
Solanum tuberosum (2)
Prunus persica (1)
Malus x domestica (2)
Cucumis sativus (1)
Eucalyptus grandis (5)
Aquilegia coerulea (1)
Theobroma cacao (3)
Gossypium raimondii (1)
Manihot esculenta (1)
Vitis vinifera (2)
Cicer arietinum (1)
Cicer arietinum (2)
Medicago truncatula (2)
Glycine max (2)
Glycine max (3)
Eucalyptus grandis (8)
Eucalyptus grandis (7)
Eucalyptus grandis (1)
Linum usitatissimum (1)
Linum usitatissimum (3)
Populus trichocarpa NP 157
Arabidopsis thaliana (3)
Capsella rubella (1)
Arabidopsis lyrata (2)
Brassica rapa (1)
Thellungiella halophila (2)
Triticum urartu (2)
Aegilops tauschii (2)
Hordeum vulgare (1)
Brachypodium distachyon (2)
Oryza sativa (1)
Sorghum bicolor (1)
Sorghum bicolor (2)
Zea mays (2)
Setaria italica (1)
Sorghum bicolor (3)
Panicum virgatum (2)
Setaria italica (3)
Oryza sativa (2)
Picea abies (3)
Picea sitchensis (1)
Picea abies (4)
Picea abies (2)
Selaginella moellendorffii (1)
Selaginella moellendorffii (2)
Arabidopsis lyrata (3)
Arabidopsis thaliana (2)
Capsella rubella (2)
Brassica rapa (3)
Brassica rapa (2)
Thellungiella halophila (1)
Arabidopsis thaliana (1)
Arabidopsis lyrata (1)
Capsella rubella (3)
Populus trichocarpa NP 870
Linum usitatissimum (2)
Populus trichocarpa NP 880
Gossypium raimondii (3)
Theobroma cacao (5)
Theobroma cacao (4)
Populus trichocarpa NP 860
Ricinus communis (1)
Prunus persica (2)
Solanum tuberosum (1)
Solanum lycopersicum (1)
Mimulus guttatus (2)
Vitis vinifera (3)
Carica papaya (1)
Picea abies (1)
76/1.0
96/1.0
-/0.93
-/0.91
-/0.82
99
98
94
-/0.93
-/0.93
89
94
64
74
96
92
98
1
99/1.0
84
93
99/1.0
-/0.99
92/1.0
-/0.99
-/1
77/1.0
-/0.83
61/0.99
93
-/0.89
78/0.90
-/0.89
-/0.79
88/1.0
Group I
Oceanobacillus sp Ndiop (1)
Paenibacillus elgii (1)
Clostridium saccharolyticum (1)
Bacterial outgroup
0.2
Inferred # of substitutions per site
