# Supplementary material for: Elucidating the evolutionary history and expression patterns of nucleoside phosphorylase paralogs (vegetative storage proteins) in Populus and the plant kingdom
Source: BMC Plant Biol. 2013 Aug 19;13:118. doi: 10.1186/1471-2229-13-118 (PMC3751785; doi:10.1186/1471-2229-13-118)
Supplement: Additional file 8: Figure S2 — Alignment of motifs predicted by MEME for bacterial and plant nucleoside phosphorylase (NP) proteins. Bacterial amino acid sequences were retrieved from the Conserved Domain Database (CDD, http://www.ncbi.nlm.nih.gov/Structure/cdd/cdd.shtml) from the NP family (COG0775) and plant amino sequences were retrieved from Phytozome (http://www.phytozome.net). [file 1471-2229-13-118-S8.pdf]

Alignment of motif sequences predicted by MEME from plant and non-plant (bacterial) NP sequences

```
                                                     20
CDDnonplant1     - - - - - - - - - - - - - - - - - - - - - - - - V A M - - P
CDDnonplant2     - - - - - - - - - - - - - - - - - - - - - - - E L A Q - - M
CDDnonplant3     - - - - - - - - - - - - - - - - - - - - - - - - L A G M F P
CDDnonplant4     - - - - - - - - - - - - - - - - - - - - - - - - L A Q - - M
CDDnonplant5     - - - - - - - - - - - - - - - - - - - - - - - - V A G - - -
CDDnonplant6     - - - - - - - - - - - - - - - - - - - - - - - E L A G - - M
CDDnonplant7     - - - - - - - - - - - - - - - - - - - - - - - - L A G - - M
CDDnonplant8     - - - - - - - - - - - - - - - - - - - - - - - - - - - - - -
CDDnonplant9     - - - - - - - - - - - - - - - - - - - - - - - - - - - - - -
CDDnonplant10    - - - - - - - - - - - - - - - - - - - - - - - - - A G - - P
plantNPmotif1    - - - - - - - - - - - - - - - - - - - - - - V D L A G - - R
plantNPmotif2    - - - - - - - - - - - - - - - - - - - - P Y V D L A G - - R
plantNPmotif3    - - - - - - - - - - - - - - - - - - - - - Y V D L A G - - R
plantNPmotif4    - - - - - - A L L Q S G S F V P D S E L P Y V D L A G - - R
plantNPmotif5    - - - - - - - - - - - - - F V P D - - - P Y V D L A G - - R
plantNPmotif6    - - - - - - - - - - - - - F P D S - - - P Y V D L A G - - R
plantNPmotif7    - - - G T P E V R Q H V F W V P V D S S W Y L A T K L - - E
plantNPmotif8    P V Y G T P E V R Q H V F W V P V D S S W Y L A T K L - - E
plantNPmotif9    - - - - - - - - - - - - - F V P D S E L P Y V D L A G - - R
plantNPmotif10   - - - - - - - - - - - - - - - - - - - - - - - - - - - - - -
                 P V Y G T P E V R Q H V F F V P         P Y V D L A G M F

                                     40                                      60
CDDnonplant1     - A F Y L G K L - G K - E V V L V N S G - I G - K V N A A L
CDDnonplant2     - P F Y H G K L - G K - E V V L L - S G - I G - K V N A A L
CDDnonplant3     - A Y Q H G K L - G K - E V V L L - S G - I G - K V N A A L
CDDnonplant4     - P A Y H G K L - G K - E V V L L - S G - I G - K V N A A L
CDDnonplant5     - Q F Y A G H L - G K - E V V L L T S G N I G - K V N A A L
CDDnonplant6     - P A Y H G K L - G K - E V V L L - S G - I G - K V N A A L
CDDnonplant7     - P A Y H G K L - G K - E V V L L - S G - I G - K V N A A L
CDDnonplant8     - - - - - - - - - - - - D L - - - - - - - - - - K V R T G L
CDDseqmotif9     - - - - - - - - - G K - E V V L V N S G - I G - K V N A A L
CDDnonplant10    - A F Y L G K L - G K G E V V L V N S G - I G - K V N A A L
plantNPmotif1    - R F R I G T I - G K - K V I Y V M T G - L S - M L N A A I
plantNPmotif2    - R F R I G E T I G K - K V I Y V M T G - L S - M L N A A I
plantNPmotif3    F R F R I G T I - G K - K V I Y V M T G - L S - M L N A A I
plantNPmotif4    - R F R I G T I - G K - K V I Y V M T G - L S - M L N A A I
plantNPmotif5    - R F R I G T I E G K - K V I Y V M T G - L S - M L N A A I
plantNPmotif6    - R F R I G T I - G K - K V I Y V M T G - L S - M L N A A I
plantNPmotif7    - D L K L E Q C V N S - T T C L P R T P K V V R G L R G S S
plantNPmotif8    Q D L K L E Q C V N S - T T C L P R T P K V V R G L R G S S
plantNPmotif9    - R F R I G T I - G K - K V I Y V M T G - L S P M L N A A I
plantNPmotif10   - - - - - - - - - - - - - - - - - - - - - - - - - - - - - -
                     F   . G   .   G K G   V . L V M . G K . . R K V N A A L

                                                     80
CDDnonplant1     I T T D L - L A V F K P D A V I N T G S A G G L R - S L K V
CDDnonplant2     T T T D L - I A V F K P D A I I N T G S A G G L R S S L K V
CDDnonplant3     T T T D L - I A V F K P D A I I N T G S A G G L R S S L K V
CDDnonplant4     T T T D L - I A V F K P D A I I N T G S A G G L R - S L K V
CDDnonplant5     K T T L L H L A V F K P D A V I N T G S A G G L R - S L K V
CDDnonplant6     T T T L L - L A V F K P D A V I N T G S A G G L R - S L K V
CDDnonplant7     T T T D L - I A V F K P D A I I N T G S A G G L R - S L K V
CDDnonplant8     I A S G D R F V E L S E R A L R N F P D A A V E M E G A A I
CDDnonplant9     T T D L L - A V L F K P D A I I N T G S A G G L R - S L K V
CDDnonplant10    I T T A L L A V L F K P D A V I N T G S A G G L S - Q L K V
plantNPmotif1    T T Q L L - L D L F D V K G I V H Y G I A G N A N P S L S I
plantNPmotif2    T T Q L L - L D L F D V K G I V H Y G I A G N A N P S L S I
plantNPmotif3    T T Q L L - L D L F D V K G I V H Y G I A G N A N P S L S I
plantNPmotif4    T T Q L L - L D L F D V K G I V H Y G I A G N A N P S L S I
plantNPmotif5    T T Q L L - L D L F D V K G I V H Y G I A G N A N P S L S I
plantNPmotif6    T T Q L L - L D L F D V K G I V H Y G I A G N A N P S L S I
plantNPmotif7    A D I F V D N A A Y R E F L Y K K F N V S T V D M E S A A V
plantNPmotif8    A D I F V D N A A Y R E F L Y K K F N V S T V D M E S A A V
plantNPmotif9    - T Q L L - L D L F D V K G I V H Y G I A G N A N P S L S I
plantNPmotif10   - - - - L - L D L F D V K G I V H Y G I A G N A N P S L S I
                 T T   L L   L . . F .     . I .   . G   A G . .   P S L   V

                                    100                                     120
CDDnonplant1     G D V V I S D - A Y V R H D V - D L T A F G - Y E - G Q I P
CDDnonplant2     G D V V I A D - A Y V Y H D V - D L T A F G - Y E - G Q I P
CDDnonplant3     G D V V I A D - A Y V Y H D V - D L T A F G - Y E - G Q I P
CDDnonplant4     G D V V I A D - A Y V Y H D V - D L T A F G - Y E - G Q I P
CDDnonplant5     G D V V I A D - A L V Y H D V - D L T A F G - Y E - G Q I P
CDDnonplant6     G D Y V I A D - E A Y Y H D V - D - T A F G A Y E L G Q I P
CDDnonplant7     G D V V I A D A Y V R Y H D V - D V T A F G - Y E P G Q I P
CDDnonplant8     A Q V C Y R F - G V P F V V I R A I S D K A - - D H E A K S
CDDnonplant9     G D V V V S D - A Y V Y H D V - D L T A F G - Y E - G Q I P
CDDnonplant10    G D V V I S A - E L R Y H D V - D V T A F G - Y E - G Q P P
plantNPmotif1    G D V S I P K - Y W A H T G L W N W Q R F G - D G P G D L L
plantNPmotif2    G D V T I P K - Y W A H T G L W N W Q R F G - S G P E G E L
plantNPmotif3    G D V T I P K - Y W A H T G L W N W Q R F G - S G P G D L L
plantNPmotif4    G D V T I P K - Y W A H T G L W N W Q R F G - S G P G D E L
plantNPmotif5    G D V T I P K - Y W A H T G L W N W Q R F G - S G P G D E L
plantNPmotif6    G D V T I P K - Y W A H T G L W N W Q R F G - S G P G D E L
plantNPmotif7    A L T C L S N - G V P F I V I R G L S D L A - - G G G S S -
plantNPmotif8    A L T C L S N - G V P F I V I R G L S D L A - G G - - - - -
plantNPmotif9    G D V T I P K - Y W A H T G L W N W Q R F G - S G P G D E L
plantNPmotif10   G D V T I P K - Y W A H T G L W N W Q R F G - S G I G Y L K
                 G D V   I     A   . . .   . . W   . .   F G A     P G   .

                                                    140
CDDnonplant1     G L P A - F V A D E - - - - - - - - - - - - - - - - - - - -
CDDnonplant2     G L P A - F V A D E - - - - - - - - - - - - - - - - - - - -
CDDnonplant3     G L P A V F V A D E - - - - - - - - - - - - - - - - - - - -
CDDnonplant4     G L P A - F V A D E - - - - - - - - - - - - - - - - - - - -
CDDnonplant5     G L P A - F V A D - - - - - - - - - - - - - - - - - - - - -
CDDnonplant6     G L P A - F E A D E - - - - - - - - - - - - - - - - - - - -
CDDnonplant7     A E P A - F L A D - - - - - - - - - - - - - - - - - - - - -
CDDnonplant8     F D E F L F A A K Q S A Q H V L I G I E - - - - - - - - - -
CDDnonplant9     G L P A - F V A D E - - - - - - - - - - - - - - - - - - - -
CDDnonplant10    G A P A Q F A L D E - - - - - - - - - - - - - - - - - - - -
plantNPmotif1    F G E - - F N G P Y T G E N L L G S F S Y Y T E - - - - - -
plantNPmotif2    F G S - - F N D P T R G E N L L G F V - - - - - - - - - - -
plantNPmotif3    F L E - - F N G P Y T R E N G L G S F A D Y - - - - - - - -
plantNPmotif4    A L E - - F N G - - - - - - - - - - - - - - - - - - - - - -
plantNPmotif5    F L E - - F N G D Y T G E N G - - - - - - - - - - - - - - -
plantNPmotif6    F L E - - F N G D Y T R E N G - - - - - - - - - - - - - - -
plantNPmotif7    - - - - - - - - - - - - - - - - - - - - - - - - - - - - - -
plantNPmotif8    - - - - - - - - - - - - - - - - - - - - - - - - - - - - - -
plantNPmotif9    F L E - - F N G D Y T R E N G - - - - - - - - - - - - - - -
plantNPmotif10   F G D - - Y N V P E N G D N L L G S V W Y Q P E E I F P V D
                   L   A   F   . D E T   E N   L G S     Y Y   E E I F P V D

                                    160                                     180
CDDnonplant1     - - - - - - - - - - - - - - - - - - - - - - - - - - - - -
CDDnonplant2     - - - - - - - - - - - - - - - - - - - - - - - - - - - - -
CDDnonplant3     - - - - - - - - - - - - - - - - - - - - - - - - - - - - -
CDDnonplant4     - - - - - - - - - - - - - - - - - - - - - - - - - - - - -
CDDnonplant5     - - - - - - - - - - - - - - - - - - - - - - - - - - - - -
CDDnonplant6     - - - - - - - - - - - - - - - - - - - - - - - - - - - - -
CDDnonplant7     - - - - - - - - - - - - - - - - - - - - - - - - - - - - -
CDDnonplant8     - - - - - - - - - - - - - - - - - - - - - - - - - - - - -
CDDnonplant9     - - - - - - - - - - - - - - - - - - - - - - - - - - - - -
CDDnonplant10    - - - - - - - - - - - - - - - - - - - - - - - - - - - - -
plantNPmotif1    - - - - - - - - - - - - - - - - - - - - - - - - - - - - -
plantNPmotif2    - - - - - - - - - - - - - - - - - - - - - - - - - - - - -
plantNPmotif3    - - - - - - - - - - - - - - - - - - - - - - - - - - - - -
plantNPmotif4    - - - - - - - - - - - - - - - - - - - - - - - - - - - - -
plantNPmotif5    - - - - - - - - - - - - - - - - - - - - - - - - - - - - -
plantNPmotif6    - - - - - - - - - - - - - - - - - - - - - - - - - - - - -
plantNPmotif7    - - - - - - - - - - - - - - - - - - - - - - - - - - - - -
plantNPmotif8    - - - - - - - - - - - - - - - - - - - - - - - - - - - - -
plantNPmotif9    - - - - - - - - - - - - - - - - - - - - - - - - - - - - -
plantNPmotif10   G T P E E A R Q H V F W V P V D S S W Y L A T K L E D L K
                 G T P E E A R Q H V F W V P V D S S W Y L A T K L E D L K
```
